# Immune monitoring technology primer: Single Cell Network Profiling (SCNP)

**DOI:** 10.1186/s40425-015-0075-z

**Published:** 2015-08-18

**Authors:** Rachael E. Hawtin, Alessandra Cesano

**Affiliations:** Nodality, 170 Harbor Way, South San Francisco, CA 94080 USA; AC Consulting, Redwood Shores, CA 94065 USA

## Description of the technology

Understanding a patients’ immune status not only from immune cell phenotyping, but also through analysis of *functional signaling capacity,* enables the generation of a more comprehensive understanding of the complex mechanisms responsible for immunological tolerance in cancer, and generates data that is complementary to other non-functional phenotypic data sets such as immunohistochemical profiling and genomic analyses. Single cell network profiling (SCNP) is a technology that quantifies functional immune signaling capacity and connectivity at a systems biology level. The technology is based on multiparametric flow cytometry that simultaneously quantifies in multiple and rare immune cell subsets, without the need for physical separation, both extracellular surface markers and changes in intracellular signaling proteins in response to extracellular modulators. Quantifying modulated signaling across a panel of modulators (e.g., IFNα, IFNγ, IL-4, IL-10, IL-27, anti-CD3 etc.) and intracellular signaling pathways identifies the functional capacity of the signaling network which cannot be assessed by measuring basal (unmodulated) signaling alone. A signaling node is defined as the combination of the extracellular modulator with the intracellular readout. For example TLR4 - > p-Erk defines one signaling node in which TLR4 modulation is quantified through the increase in p-Erk levels as compared to the unmodulated reference. Typically 3 nodes are captured simultaneously per well across multiple immune cell subsets of interest (e.g., TLR4 - > p-Erk, p-S6, IkB).

**Fig. 1:**
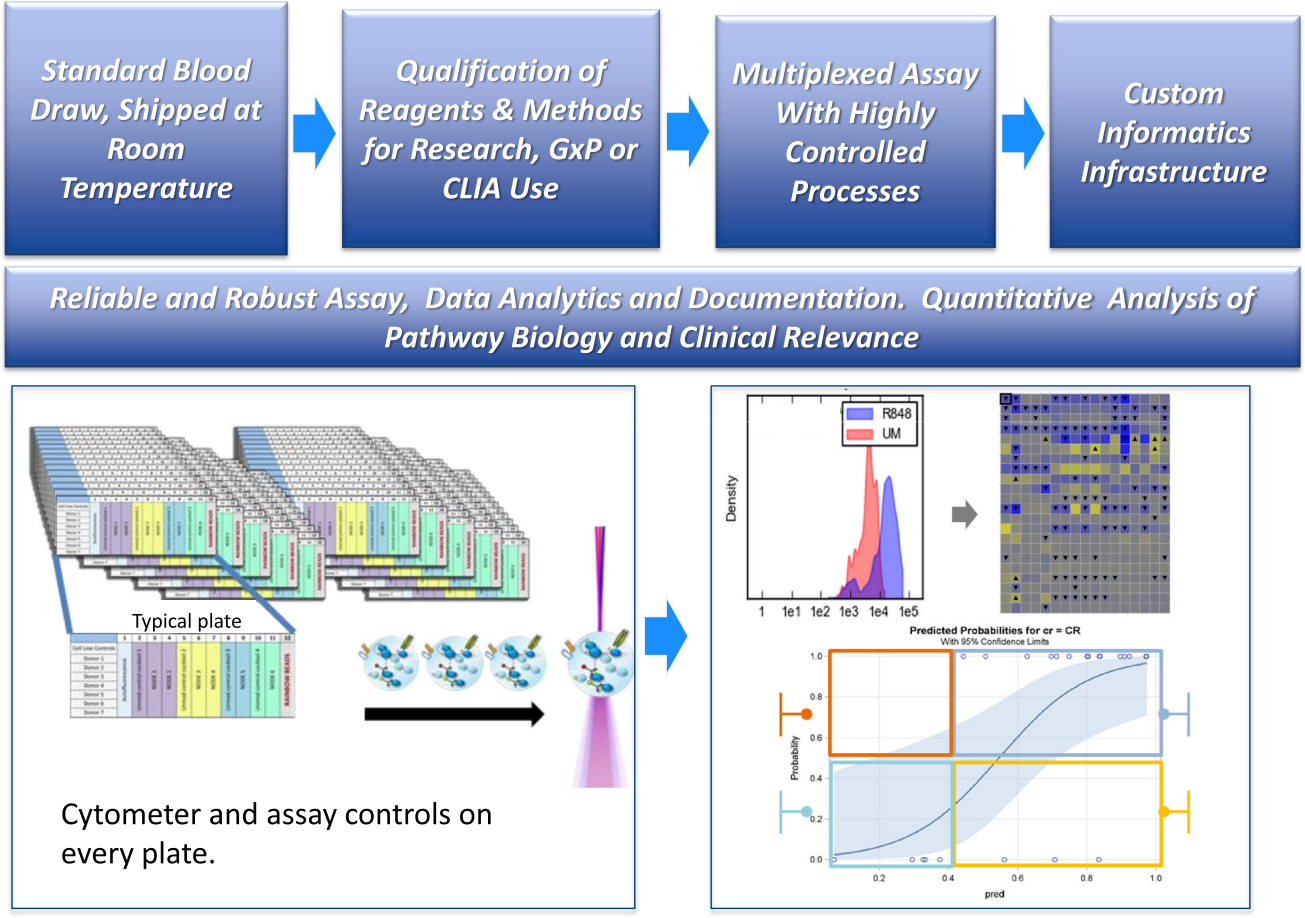
SCNP Process. The SCNP process is coordinated from sample collection through to data analysis and visualization. Sample collection requires standard sample draw into sodium-heparin coated tubes and overnight shipment at ambient temperature. Reagents and methods for the laboratory assay are qualified and controlled for research, GxP or CLIA use as appropriate. The laboratory assay is controlled and multiplexed across 96 well plates to enable broad biological analysis of each donor sample, with normalization across plates, instruments and time. A custom informatics infrastructure ensures reliable and efficient data tracking and maintenance of data integrity. Multiple metrics are established for analysis of signaling magnitude, cell population-based responses and signaling inhibition

**Fig. 2:**
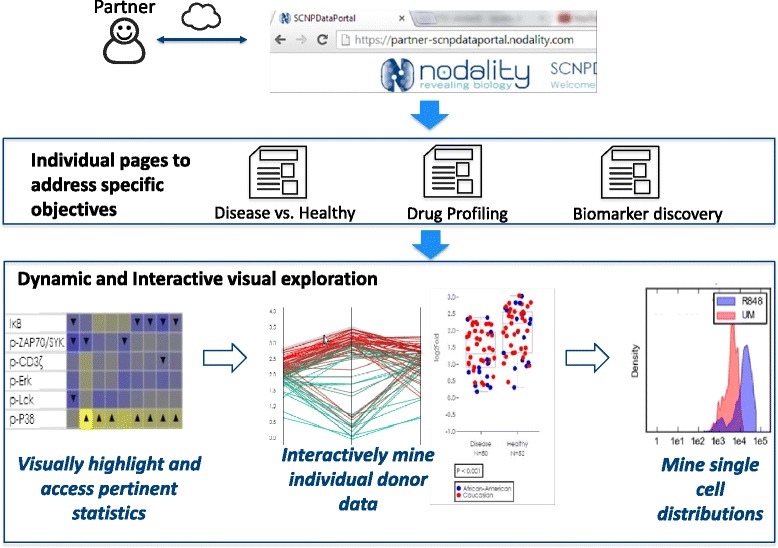
Data analysis. The cloud-based SCNPDataPortal enables investigator analysis of the data set in an interactive manner. Individual web pages present data that address the specific objectives for each study. In the example shown these pages enable the analysis of; disease compared to healthy donor signaling; interrogation of the mechanism of action (MOA) of a candidate molecule or therapeutic; analysis of biomarkers for pharmacodynamic studies, patient selection / stratification and / or toxicity. Visualization options include heatmaps providing an overview of the data and serving as a platform for deeper data mining. Parallel plots provide a means to identify donor subgroups, with each line representing a donor sample with the associated signaling across multiple nodes and cell subsets. In the example shown, two donor subgroups are identified by analysis of signaling in 3 node/cell subset combinations. Box-and-whisker plots enable the comparison of the range of signaling between donor subgroups for selected nodes/cell subsets, with the coloring of each donor data point based upon demographic or clinical criteria. In the example shown, signaling is compared in disease vs. healthy donor samples, with color coding for African American (blue) or caucasian (red) donor samples. Clicking on the individual donor data enables interrogation of the single cell distributions for each data point

The application of SCNP to clinical decision-making requires the generation of high-content SCNP assays with robust, accurate, quantifiable and reproducible results across time, operators and instruments. Each of the procedural steps associated with an SCNP assay, including pre-analytical sample handling, assay execution and reagents, data acquisition and analysis and the generation of metrics, have been validated [1] (Figs. 1 and 2).

Experimental assay setup is performed using proprietary software which enables experimental design/96-well plate layouts and data capture to be contiguously linked, ensuring that data from each well is correctly assigned. The laboratory execution can be performed on as many as 30 samples assayed for up to 40 wells (approximately 200–500 SCNP dimensions comprising modulator/inhibitor/intracellular readout/cell subset combinations) in 2 to 3 days depending on the kinetic time points. A statistical analysis plan (SAP) is drafted for all studies beyond the exploratory phase, based upon clearly stated objectives. For the identification of clinically validated classifiers the time frame for assay development and validation is comparable to that of other technologies (e.g., genomics, IHC) due to the requirements for statistical powering and for verification and validation in independent sample sets.

## Type of data obtained/readout

Currently the use of 10 cytometer channels is routine, which includes 8 colors plus forward and side scatter. The biological reads labeled per well can be expanded by combining in one channel markers that are mutually exclusively expressed on immune cells, for example CD4 and CD20. SCNP further increases dimensionality of data obtained per sample via multiplexing as illustrated in Fig. 1, using controls for both assay and cytometer performance. Raw data (median fluorescence intensities) are converted to calibrated metrics using control rainbow calibration particles on each 96-well plate. This allows for normalization of readouts across 96 well plates, across flow cytometry instruments, and for the same instrument over time. Captured data includes quantification of cell subset frequencies and specific intracellular readouts for each of the cell subsets in both the basal (unmodulated) and modulated state. In addition, various aspects of modulated signaling in each cell subset, and/or signaling inhibition by *in vitro* drug exposure, are captured by metrics that are computed by comparing data for cells subject to different conditions. The “Fold” metric is applied to measure magnitude of the responsiveness of a signal in a specific cell population relative to the unmodulated reference. The proportion of a cell population that is responsive to modulation is measured by the Uu (rank based metric based on Mann–Whitney U statistic) metric. Similarly, inhibited signaling is captured using both magnitude and population-based metrics.

For each study, node metric comma separated value (CSV) data files are generated which capture signaling across cell subsets, linked with the complete meta data annotations. These data can be integrated with data from other platforms as appropriate and analyzed using standard statistical tools. Analyses can address multiple objectives including the development of multi-variate predictors of response or prognosis, clustering, quantification of signaling network function, quantification of drug activity across nodes and immune cell subsets (including IC50 determination). More comprehensive and interactive mining of these multidimensional data sets is enabled via an interactive web-based data portal (Fig. 2, concept demonstrated as a video in [2]). The data portal enables online data analysis and visualization, with associated statistical annotations.

## Limitations of the approach

SCNP requires live cells in suspension, and therefore is not amenable to analysis of immune cells in flash frozen or FFPE tissue samples. While broad profiling of signaling per sample has been enabled by multiplexing and an extensive array of biology has been established using this technology, the assay is dependent upon the availability of antibodies suitable for flow cytometry. Analysis of immune signaling associated with antigen specificity has not been established. It remains to be seen whether the technology can be transferred to tumor cells isolated from solid tissue.

## Advantages of the approach

One of the major advantages of this technology in the context of immuno-oncology is the ability to monitor cellular functional capacity without physical cell isolation. This enables the detection and monitoring of immune signaling and communication within the complex and interlocked immune system. Interrogating the frequency and signaling capacity of immune cells, and the effects of targeted therapeutics following either clinical administration or *in vitro* exposure, can inform on disease and drug mechanism, combination strategies, and identify biomarkers for pharmacodymanic or patient stratification that are otherwise inaccessible using less sensitive or non-functional analyses [3–15]. The standardization of the SCNP process, combined with the ability to interrogate signaling in rare cell subsets enables the quantification of clinically relevant yet subtle signaling shifts that would be otherwise undetectable using cell-averaging approaches, or technologies which are not highly standardized or reproducible.

## Types of samples needed and special issues pertaining to samples

Appropriate pre-analytic sample manipulation is crucial. SCNP has been used successfully with whole blood, PBMC, BMMC [1] lamina propria mononuclear cells isolated from human gut tissue, pleural fluids and bladder washings. Sample handling procedures are consistent with broadly used protocols, involving sample draw into sodium heparin coated tubes, shipment at ambient temperature and processing using Ficoll separation, typically within 24 h of sample draw [16].

## Level of evidence

Over 30 manuscripts and 70 abstracts have been published using this platform. The analytic and clinical validity is presented in many of these, with example references below [1, 3–16]. The assay is conducted as appropriate in a Research, GLP/GMP or CLIA certified laboratory. Clinically validated classifiers for the prediction of response/non response to standard induction therapy in front line AML in both the elderly and pediatric populations have been generated [3, 4]. Successful application of SCNP to multiple stages of drug development has been reported, encompassing mechanistic studies on molecules in early development through to therapeutics in clinical development or use [13–15]. Signaling in primary patient samples from metastatic melanoma, AML, CLL, MDS, rheumatoid arthritis (RA) and systemic lupus erythematosus (SLE) donors is routinely analyzed.
